# Isolation and worry in relation to gambling and onset of gambling among psychiatry patients during the COVID-19 pandemic: A mediation study

**DOI:** 10.3389/fpsyg.2022.1045709

**Published:** 2022-12-22

**Authors:** David Forsström, Philip Lindner, Kristoffer N. T. Månsson, Olivia Ojala, Maria Hedman-Lagerlöf, Samir El Alaoui, Alexander Rozental, Johan Lundin, Simon Jangard, Shervin Shahnavaz, Karolina Sörman, Tobias Lundgren, Nitya Jayaram-Lindström

**Affiliations:** ^1^Department of Clinical Neuroscience, Centre for Psychiatry Research, Karolinska Institutet, Stockholm Health Care Services, Stockholm, Sweden; ^2^Department of Psychology, Uppsala University, Stockholm, Sweden; ^3^Department of Psychology, Stockholm University, Stockholm, Sweden; ^4^Centre for Dependency Disorders, Stockholm Health Care Services, Stockholm, Sweden; ^5^Department of Clinical Neuroscience, Karolinska Institutet, Stockholm, Sweden; ^6^Division of Psychology, Department of Clinical Neuroscience, Karolinska Institutet, Stockholm, Sweden

**Keywords:** COVID-19, gambling, isolation, worry, common mental disorders

## Abstract

When the COVID-19 pandemic started spreading globally, there was a fear that addictive behaviors would increase due to changes in everyday life caused by restrictions due to COVID-19. Studies were carried out to explore if this was true for gambling, typically revealing no overall increase in gambling behavior, although individuals who had previous experience with gambling problems were more likely to increase gambling during the pandemic. However, these studies only included individuals with previous gambling problems. It remains unknown whether other vulnerable groups, such as individuals with common mental disorders increased their gambling. This study aimed to explore the level of gambling problems among individuals with a history of mental disorders, namely, (i) pre-pandemic gamblers and (ii) pandemic-onset gamblers. Furthermore, we explored if worry and isolation mediate gambling and problem gambling. The data were analyzed using descriptive statistics and a structural equation model to investigate mediation. The results showed a high prevalence of at-risk and problem gambling in both groups. The pre-pandemic gamblers had a high level of at-risk and problem gambling. Furthermore, the individuals that started to gamble during the pandemic had an even higher degree of at-risk and problem gambling. The mediation showed that the onset of gambling was linked with the worry of COVID-infection and that worry predicted the level of gambling problems. This study highlights that vulnerability factors, isolation, and worry can be triggers for individuals with common mental disorders to engage in gambling as well as the importance of screening this population for gambling problems.

## Introduction

The prevalence of at-risk and problem gambling is 1.3% in Sweden (Hofmarcher et al., 2020). In Sweden, approximately 160,000 significant others are affected by individuals that are at risk for developing gambling problems (Hofmarcher et al., 2020), making this a public health issue. Worldwide, the prevalence rate ranges from 0.12 to 5.80% (Calado and Griffiths, 2016), making it a serious public health concern.

During the early stages of the COVID-19 pandemic, when social distancing and restrictions were imposed around the world, concerns were raised about how the resulting changes in everyday life would have an impact on public health, including gambling habits (Håkansson et al., 2020; Stark and Robinson, 2021). Research has shown that previous global crises (e.g., the economic crises in Iceland and Greece) led to increased gambling (Olason et al., 2015; Economou et al., 2019), and during the early periods of the COVID-19 pandemic, it appeared to increase mental health problems (Brodeur A. et al., 2021). However, several populations remain to be investigated to understand the ramifications of COVID. Several reviews have addressed different types of online behaviors during the pandemic. Internet use, smartphone use in general, Internet gaming, and the use of pornography increased during the pandemic (Masaeli and Farhadi, 2021). This review, which specifically included nine gambling studies that covered the first wave of the pandemic between March 2020 and May 2020, found that there was an overall decrease in gambling. High-risk gamblers were, however, more likely to gamble more, migrate to online gambling, and had a higher degree of anxiety and depression during the pandemic (Masaeli and Farhadi, 2021). This tendency, as found in the review, can potentially be explained by the fact that sports events were canceled, and betting opportunities disappeared as a consequence of the pandemic. In contrast to this, other studies have found that individuals who already had a high degree of gambling prior to the pandemic started to gamble more (Brodeur M. et al., 2021; Hodgins and Stevens, 2021). This increase is not large enough to compensate for the overall decrease, however, resulting in two opposite trends in gambling activity. Hodgins and Stevens (2021) included 17 studies in their review (including non-peer-reviewed governmental reports) and found that, overall, gambling decreased, as measured by total spending on gambling. This was prevalent in the group that did not have gambling problems (Hodgins and Stevens, 2021). However, in this case, the studies that were included give a contradictory account of the gambling situation. Some reported an increase in online gambling, while others observed no change. In part, the contradictory results could be explained by the use of populations with different characteristics, e.g. the age of the sample and the level of gambling problems among the populations included (Hodgins and Stevens, 2021). Furthermore, another confounding aspect of the change in gambling patterns is that the pandemic resulted in changes in legislation for some jurisdictions, among them was Sweden (e.g., deposit limits and other actions that regulated online gambling), which have impacted the way individuals gamble in different jurisdictions (Capriulo et al., 2021).

Recently, published studies covering the subsequent waves (wave two between September and December 2020 and wave three between December 2020 and April 2021) of the pandemic further clarifies the effect of COVID-19 on gambling, demonstrating decreased gambling overall, and an increase among vulnerable populations. Lugo et al. (2021) exhibited results along this line. In this study, vulnerable populations mean men that are between the ages of 18 and 25, which is a group that is vulnerable when comes to the risk of developing gambling problems. However, a few studies did report an increase in gambling behaviors in relation to the pandemic, specifically in groups with a history of previous gambling (Emond et al., 2022). This is comparable to the results in the study by Håkansson and Widinghoff (2021), where at-risk and problem gamblers were more likely to gamble. However, only 6% (*n* = 114) of the sample in that study population did report increased gambling Håkansson and Widinghoff (2021). Furthermore, an additional study found that the restriction of gambling neither leads to an increase nor decrease in gambling over time among regular gamblers (Black et al., 2022). A study carried out in Poland found that sports bettors did migrate to other forms of gambling when sporting events ceased, which in turn could result in an increase in problem gambling for that group, although the long-term effects remain to be investigated (Nosal and Lopez-Gonzalez, 2021). Håkansson et al. (2021) investigated if there were any changes in the use of the Swedish National Self-Exclusion Registry (individuals who gamble can exclude themselves from licensed gambling sites in Sweden *via* their social security number). The study did not find any divergent changes in self-exclusion patterns in Sweden. Results from a study carried out in Finland showed that gambling behavior as well as the demand for treatment services for gambling disorders decreased (Marionneau and Järvinen-Tassopoulos, 2022). While several reviews and studies have shown that total gambling activity decreased during the first phase of the COVID-19 pandemic between March 2020 and May 2020, consistent with closed gambling locales and lost betting opportunities, online casino gambling, however, showed mixed results concerning a decrease in gambling, and this was moderated by subgroup based on the severity of gambling problems (Brodeur M. et al., 2021; Hodgins and Stevens, 2021).

At present, a clear interpretation of the mixed results regarding online gambling is lacking. It could be driven by individuals that gambled before the pandemic, most of whom can be expected to engage in both betting and casino games (Auer et al., 2020), changing their betting-casino ratio as betting opportunities were lost, or new gamblers engaging in casino games due to, for instance, anxiety or even boredom caused by the isolation during the COVID-19 outbreak. What seems clear, however, is that individuals with pre-pandemic gambling problems were more at risk of developing more severe gambling problems during the COVID-19 pandemic.

Most of the studies to date have targeted populations that were gambling before the pandemic. Research is lacking regarding gambling patterns in other vulnerable populations, such as individuals with low socio-economic status and individuals with common mental disorders with or without other substance abuse disorders. For example, individuals with common mental disorders may have the propensity to either begin or increase gambling, as a consequence of increased isolation and worry. One study explored the prevalence rate of gambling problems in a psychiatric population (pre-pandemic study) and found higher rates than in the overall population (Vita et al., 2021). Several studies have investigated comorbid conditions alongside gambling. One review found that 37.4% of individuals with gambling disorder also had an anxiety disorder (Lorains et al., 2011). Also, one study found a link between problem gambling and worry in young adults (Slutske et al., 2005). Both studies indicate that there is a link between anxiety, worry, and problem gambling. However, since only a few studies have examined the link between common mental disorders and gambling problems in that specific population, there is a need for more research investigating this relationship and how this group has changed their gambling patterns.

Our current study aimed to examine levels of gambling and gambling problems according to the results of the Problem Gambling Severity Index (Ferris and Wynne, 2001) during the COVID-19 pandemic in a population with common mental disorders and compare those who gambled before the pandemic with those that started gambling during the pandemic. The relationship between gambling problems and worry, as well as isolation due to the pandemic, will also be assessed.

## Materials and methods

### Ethics

The Swedish Ethical Review Authority approved this study (ID 2020-02798). Electronic informed consent was collected at the beginning of the online questionnaire. The data collection and the overall study were conducted in accordance with the Helsinki declaration.

### Procedure and sample

The described analyses are part of a larger cohort study focusing on the impact of COVID-19 among patients with common mental disorders in Sweden (Rozental et al., 2022). Data collection for this cohort study began in July 2020 (shortly after the first disease wave in Sweden) and ended in June 2021. An open online survey was used for data collection,^1^ which also contained study information. Refer to Rozental et al. (2022) for the inclusion criteria. The instruments used in the survey were taken from the RAMP study to be able to compare the results.^2^ Only a selection of the instruments from the survey was used in our study. To be able to recruit a large sample, comprehensive information campaigns were carried out in collaboration with non-governmental organizations focused on mental health, psychiatric clinics, and advertising on social media such as Facebook and Instagram. Television and radio presentations were also a part of the information campaign.

The survey asked whether the respondent had gambled during the month preceding completing the survey. An additional question regarding if the respondent gambled before the pandemic was also posed. Respondents that had gambled before the pandemic were labeled pre-pandemic gamblers, and respondents that started during the pandemic but not before the pandemic were labeled pandemic-onset gamblers.

In total, 6,095 respondents were included. Of those, 510 respondents agreed to have gambled during the past-month, of which 418 had gambled before the pandemic and 92 respondents started gambling during the pandemic. This provided 80% power to detect a between-group difference corresponding to *d* > 0.32 (a small-medium effect size), which was deemed to be in the range of clinical relevance.

### Measures

#### Demographics

Several different demographics were collected [refer to Rozental et al. (2022) for a complete description of the whole sample]. Refer to Table 1 for gender distribution, mean age, and marital status for the complete study population and for those individuals who reported having gambled, respectively.

**TABLE 1:** Sample characteristics by subgroup.

Variable	Full sample (*n* = 6095)	Pandemic-onset gamblers (*n* = 92)	Pre-pandemic gamblers (*n* = 418)
**Sex**			
Man	*n* = 1490 (24.4%)	38 (41.3%)	171 (40.9%)
Woman	*n* = 4304 (70.6%)	53 (57.6%)	235 (56.2%)
Non-binary	*n* = 301 (5%)	1 (1.1%)	12 (2.9%)
Mean age (SD)	35.05 (12.1)	34.7 (11.5)	41.9 (11.9)
**Marital status**			
Single	*n* = 2632 (43.2%)	40	163
Living apart but are together	*n* = 736 (12.1%)	14	43
Cohabitation/Married	*n* = 2443 (40.1%)	32	181
Divorced	*n* = 255 (4.2%)	4	26
Widow/widower	*n* = 29 (0.5%)	2	5

#### Social isolation

Social isolation was measured by one item that asked if one felt more isolated after the onset of COVID-19. The question was as follows: “How isolated do you feel now in comparison to how you felt before the pandemic?” The answers ranged from “less isolated,” “same as before,” and “more isolated.” The item was recoded in our study and was split into two categories, i.e., less or no change and more isolated. The variable was recoded into a binary variable (no change or less isolated and more isolated) in order to examine the impact of increased isolation on gambling problems.

#### COVID worry

The questionnaire was designed to measure worry in relation to the COVID-19 pandemic and was created by Repeated Assessment of Mental Health in Pandemics. The instrument contains 19 items. The answers range from “not at all worried” to “extremely worried.” Cronbach’s alpha in the subsample that reported any gambling was α = 0.84, and the bootstrap confidence interval (CI) was 95% (0.82–0.86). The sum of the items was used as a variable in the study.

#### Lifetime psychiatric diagnoses

The participants were asked if they had been diagnosed with common psychiatric diagnoses, e.g., generalized anxiety disorder (refer to Supplementary material for a complete list). The number of endorsed diagnoses was added up and constituted the variable used.

#### Problem gambling

The Problem Gambling Severity Index (PGSI) was derived from the Canadian Problem Gambling Inventory (Wynne, 2003), which measures the signs and consequences of problem gambling. The self-report has nine items, e.g., “Have you felt guilty about the way you gamble or what happens when you gamble?” and “Have you needed to gamble with larger amounts of money to get the same feeling of excitement?,” and is scored on a 0–3 rating scale. The total score of the PGSI ranges from 0 to 27 (Ferris and Wynne, 2001). A score of eight and over indicates high risk and problem gambling; 3–7 indicates at-risk for problem gambling and can be seen as medium risk; and 1–2 indicates low risk. The PGSI has a test-retest reliability of 0.78 and Cronbach’s α of 0.84 (Ferris and Wynne, 2001). Cronbach’s α for the subsample that reported any gambling was α = 0.95, and the bootstrap CI was 95% (0.94–0.96). Only the respondents that answered yes to have gambled during the latest month could answer the PGSI.

### Statistical analyses

To examine associations between pre-pandemic vs. pandemic-onset gambling, current gambling problems, the total number of lifetime psychiatric diagnoses, and isolation, mediation analyses were conducted within a structural equation modeling framework. The mediation models featured a direct path between pandemic-onset vs. pre-pandemic onset gambling (binary) and the PGSI score (numeric), with an indirect path *via* COVID-related worry (numeric). This model was run first using the entire subsample of past-month gamblers (who provided complete PGSI ratings) and then again split by reported increased social isolation (*n* = 123) or not (*n* = 387). Analyses were conducted using the lavaan R package (Rosseel, 2012), with bootstrapped CIs (*k* = 5,000).

## Results

### Prevalence of at-risk and problem gambling

High rates of at-risk and problem gambling were present in both subsamples. Among pandemic-onset gamblers, 16.30% scored above eight on the PGSI and thus considered problem gamblers, while 14.10% were at-risk. Among pre-pandemic gamblers, 8.80% were problem gamblers and 11.00% were at-risk gamblers. Overall, the prevalence of current problem gambling was 0.85 and 0.97% at-risk gamblers in the entire sample of 6,095 individuals. Furthermore, only 8.40% of the sample gambled.

### Associations with pandemic-onset gambling

Among the subsample who reported any past-month gambling (*n* = 510), those who reported no pre-pandemic gambling reported significantly higher on both gambling problems according to PGSI (*B* = −1.43, *SE* = 0.59, *p* = 0.0153) and COVID worry scores (*B* = −8.12, *SE* = 1.74, *p* < 0.001). Moreover, pandemic-onset gamblers also reported a greater frequency of self-reported lifetime psychiatric diagnoses and isolation during the pandemic (88 vs. 74%, *OR* = 0.37, *p* = 0.002).

The initial mediation model revealed a significant indirect path between pandemic-onset gambling and gambling problems (*B* = −0.54, *SE* = 0.19, *p* = 0.004) such that pandemic-onset gambling was associated with greater COVID worry (*B* = −8.12, *SE* = 1.73, *p* < 0.001) and COVID worry in turn predicted the PGSI scores (*B* = 0.07, *SE* = 0.02, *p* < 0.001), rendering the direct path between pandemic-onset gambling and PGSI scores insignificant (*B* = −0.89, *SE* = 0.65, *p* = 0.17). A similar mediation model only including those who reported being more isolated (*n* = 387) vs. the same or less isolated (*n* = 123) during the pandemic revealed that the indirect path was significant only among those more isolated (*B* = −0.52, SE = 0.22, *p* = 0.017) but not those the same or less isolated during the pandemic (*B* = −0.39, *SE* = 0.33, *p* = 0.24), refer to Table 2. To estimate whether there was any confounding effect of the year-long recruitment window, the significant mediation model (including only those experiencing more isolation) was a rerun, now stratified by data of recruitment, defined as either early or late (chosen due to bimodal distribution of completed surveys during recruitment window, with median-split subgrouping of all who answered the PGSI). Comparing confidence intervals of the respective indirect effects revealed significant overlap (−1.89 to −0.12 and −0.92 to −0.02), suggesting similar mediation regardless of when participants were recruited.

**TABLE 2:** Mediation results.

Model	Direct path (PGSI ∼ pandemic-onset gambling)	A path (COVID worry ∼ pandemic-onset gambling)	B path (PGSI ∼ COVID worry)	Indirect path (A × B)
Full subsample (*n* = 510)	B = −0.89, SE = 0.64, *p* = 0.167	*B* = −8.12, SE = 1.73, *p* < 0.001	B = 0.067, SE = 0.018, *p* < 0.001	*B* = −0.54, SE = 0.19, *p* = 0.005
Subsample reporting more social isolation (*n* = 387)	B = −0.94, SE = 0.71, *p* = 0.189	B = −7.23, SE = 1.92, *p*<0.001	B = 0.07, SE = 0.02, *p* = 0.001	B = −0.52, SE = 0.22, *p* = 0.018
Subsample not reporting more social isolation (*n* = 123)	B = −0.70, SE = 2.00, *p* = 0.725	B = −6.80, SE = 4.22, *p* = 0.11	B = 0.06, SE = 0.03, *p* = 0.035	B = −0.39, SE = 0.32, *p* = 0.228

Pandemic-onset gambling variable binary, remaining numeric.

## Discussion

The results of the present study show a high rate of the problem and at-risk gambling in individuals with existing common mental disorders, both in pre-pandemic gamblers and pandemic-onset gamblers. In the latter group, increased COVID worry and isolation in turn increased the level of problem gambling.

In comparison with results from the Swedish Longitudinal Gambling study, which demonstrated a prevalence of at-risk and problem gambling at 1.30% (Folkhälsomyndigheten, 2022), the prevalence of at-risk and problem gambling was higher in our sample of individuals with a self-reported lifetime history of psychiatric disorders (1.83%). However, an important difference is that only 8.40% of our sample had gambled the last month, as compared with a much higher number in the general population, i.e., approximately 65% according to Folkhälsomyndigheten (2022). The prevalence rates in our study are high in relation to similar international studies (Williams et al., 2012; Calado and Griffiths, 2016). In pandemic-onset gamblers, however, the prevalence rate of gambling during the pandemic is similar to the prevalence rate found in a review focused on homeless individuals (Vandenberg et al., 2022), indicating that vulnerable populations of various kinds are more at risk when it comes to problem gambling.

Pandemic-onset gamblers had a higher degree of problem gambling compared to pre-pandemic gamblers. They also had a higher degree of worry, loneliness, and a number of self-reported psychiatric diagnoses. Previous research has suggested that individuals that were at-risk or gambled on a problematic level before the pandemic had an increase in their gambling problems during the pandemic (Brodeur M. et al., 2021; Hodgins and Stevens, 2021). A similar vulnerability seems to be present for individuals with a mental disorder, for both new and pre-pandemic gamblers. This is in line with a study investigating gambling among individuals with mental disorders (Vita et al., 2021), which found a higher prevalence of at-risk and problem gambling among psychiatric patients compared to a community sample. Furthermore, there seems to be a link between at-risk gambling and a higher degree of loneliness and worry due to the pandemic. The results showed that worry plays a central role when it comes to gambling problems and also that more psychiatric diagnoses are related to gambling problems. Since the sample has more women than men, this might be consistent with a review that indicated that women gamble to avoid negative emotions such as everyday stress and psychological comorbidity (Shannon et al., 2017). Also, gambling due to social isolation is an aspect of the reasons why women gamble (Shannon et al., 2017). This could in part explain the results of mediation analysis. Gambling could have been used as a maladaptive coping strategy to deal with feelings of isolation and worry. The problems with gambling that arose as a consequence of this might have been worsened due to a lack of social support in this group. The high prevalence of at-risk and problem gambling in the entire sample of 510 respondents indicates a need for screening and prevention strategies that target gambling for psychiatric populations. A previous study also found elevated prevalence rates in the psychiatric population (Vita et al., 2021), indicating that this might be a pattern across different psychiatric populations.

### Clinical implications

The fact that worry was a mediator for the gambling problem has implications for treatment and prevention. Screening and targeting worry in gambling populations with vulnerabilities could be a way of lessening the harm that can be present among individuals that have had the gambling problem for a long period of time. Information about telephone self-help lines and self-help groups is also needed to be distributed at outpatient psychiatric units and within primary care settings. Furthermore, treatment providers need to be alerted that a new segment of individuals and pandemic-onset gamblers with common mental disorders might request treatment in the wake of the pandemic and that their characteristics could differ from individuals usually seeking treatment. To that end, screening, assessment, and treatment models need to be adapted to encompass a high degree of psychiatric comorbidity. Given the results, more attention needs to be placed on screening women in different healthcare settings in order to detect at-risk and problem gambling.

### Implications of responsible gambling, legislation, and policy

In Sweden, regulators changed the existing framework for licensing during the pandemic, e.g., limiting bonuses and setting a cap for depositing money, for gambling companies to offer gambling. These changes sharpened the conditions for operating with a license in Sweden, and several measures to prevent at-risk and problem gambling were put in place, e.g., limiting bonus offers and the loss amount. However, these changes might not have had an effect on individuals with a high degree of vulnerability, such as multiple psychiatric disorders or a high-level gambling before the pandemic, and there is a need to investigate what effect these measures had on gambling for different groups. Furthermore, the deposit limits were so high that groups in a more vulnerable socio-economic situation with lower economic resources might not have been affected by the limit of 5,000 SEK per week (∼€470). Extra funding for treatment services might need to be added to the actions taken by the Swedish government to reduce and prevent gambling disorders.

Also, gambling companies could report the number of new customers during the pandemic along with information about the overall risk profile based on gambling data for these individuals, so it is possible to explore the risk levels of new gamblers during the pandemic. Also, if raw data on spending would be made available, researchers could analyze pandemic-onset gamblers in a more comprehensive way. Furthermore, if data are made available to researchers and the public regarding new customers and prevalence rates, this might call for new legislation, prevention, and research. Furthermore, the steps taken to reduce the risk for new gamblers with high risk should also be reported by gambling companies to be able to discern what type of prevention tools new gamblers are most likely to use and what types of interventions have an effect on decreasing time and money spent on gambling. If the available preventive strategies, mainly supplied online by gambling companies, are not effective, new strategies need to be researched and made available for at-risk customers.

### Limitations

When using a survey that is trying to capture different types of behavior, there is always a risk of self-report bias and responses being inaccurate. When it comes to the questions regarding gambling, the survey only asked if the respondents had gambled during the last month prior to the survey. This might have influenced the endorsement of gambling in the sample since some respondents might gamble less often than once a month. This could have decreased the number of participants that should have been included in the analysis.

Individuals who responded to the survey might have experienced less or more severe forms of loneliness and worry and are thus not representative of the population that has common mental disorders. This might have produced inflated results and perhaps a higher prevalence rate of at-risk and problem gambling. However, according to the baseline measurement, the participants had anxiety of differing severity (not necessarily linked with COVID), which indicates that it is similar to a mental health population, making them comparable to a psychiatric population, refer to Rozental et al. (2022) for more information.

There were a large number of women that answered the survey. One possible reason for this is that women are more prone to seek help for mental illness in Sweden (Kosidou et al., 2017). This might have produced a bias in relation to men with mental illness that are not a part of a help-seeking population, and they might therefore be missing among the respondents in the study.

The questions on pandemic worry have not been independently psychometrically evaluated. Since it has only been included in one other study to measure pandemic worry, one possible aspect is that the whole construct related to the pandemic is not covered by the question. This, in turn, could have led to an overestimation or underestimation of pandemic worry in the sample and the effect of worry in the result of the regression analysis, but since pandemic worry affected the level of gambling, a more precise measure might have increased the effect of worry in the model. Another limitation was that isolation was only measured with one item with only three different ways to answer the question. This might have restricted the experiences of isolation in the sample. Furthermore, that you cannot establish a temporal order in a mediator analysis also limits the conclusion that be drawn from the study.

### Future research

Future research should investigate longitudinal patterns among individuals that gambled before the pandemic and also the population that started gambling during the pandemic to be able to track changes in risk levels over time. Also, the use of responsible gambling features among new gamblers should be examined as well since our study showed a higher degree of severity for that group. In addition, the treatment-seeking behaviors of pandemic-onset gamblers should also be explored further to which extent they seek treatment. This is because treatment seeking among individuals with gambling disorder is low, and one study found that only 7–12% sought formal treatment (Slutske, 2006). The results also indicate the need for screening and research studies, exploring gambling for patients in primary care and outpatient psychiatric care that has a mental disorder. Future research should also focus on help-seeking among gamblers with mental health issues. Different aspects such as what treatment they received, how they managed to get treatment, and treatment satisfaction should be explored.

## Conclusion

Our study explored gambling behavior during the pandemic among individuals with common mental disorders. Our results showed that isolation and worry are related to higher degrees of problem gambling and difficulties due to gambling. The higher rate of problem gambling is in line with previous COVID-related gambling research where vulnerable populations are at increased risk. Also, the mediation analysis showed that there was a link between worry and isolation and pandemic gambling and problems due to gambling. Post-pandemic strategies are needed in order to help different types of vulnerable populations that gamble.

## Data availability statement

The raw data supporting the conclusions of this article will be made available by the authors, without undue reservation.

## Ethics statement

The studies involving human participants were reviewed and approved by The Swedish Ethical Review Authority approved the study (ID 2020-02798). The patients/participants provided their written informed consent to participate in this study.

## Author contributions

DF: conceptualization, methodology, validation, formal analysis, investigation, data curation, writing the original draft, reviewing, and editing. PL: validation, formal analysis, and writing—review and editing. KM: conceptualization, methodology, validation, investigation, writing—review and editing, and project administration. OO, MH-L, SE, AR, JL, SJ, SS, KS, and TL: validation and writing—review and editing. NJ-L: conceptualization, methodology, validation, writing—review and editing, supervision, and funding acquisition. All authors contributed to the article and approved the submitted version.

## Footnotes

1
www.psykiskohalsacovid19.se

2
http://rampstudy.co.uk/
