# Supplementary material for: Isolation and worry in relation to gambling and onset of gambling among psychiatry patients during the COVID-19 pandemic: A mediation study
Source: Front Psychol. 2022 Dec 22;13:1045709. doi: 10.3389/fpsyg.2022.1045709 (PMC9813864; doi:10.3389/fpsyg.2022.1045709)
Supplement: Supplementary file 1 [file Data_Sheet_1.PDF]

Supplementary material: list of diagnoses

Generalized anxiety
Postpartum depression
Premenstrual dysphoric syndrome
Bipolar disease
Alcohol or drug problems
Eating disorders
Anorexia nervosa
Psychosis
Schizophrenia
Neuropsychiatric disabilities (e.g. ADHD, Asperger's, autism)
Social phobia
Hypomanic bipolar or manic depression
Specific phobia (e.g. spider phobia)
Obsessive compulsive disorder
Health anxiety
Panic attacks
Agora phobia
Posttraumatic stress syndrome
Depression
